# A distinct bacterial dysbiosis associated skin inflammation in ovine footrot

**DOI:** 10.1038/srep45220

**Published:** 2017-03-24

**Authors:** Grazieli Maboni, Adam Blanchard, Sara Frosth, Ceri Stewart, Richard Emes, Sabine Tötemeyer

**Affiliations:** 1 University of Nottingham, School of Veterinary Medicine and Science, Sutton Bonington, United Kingdom; 2 Department of Biomedical Sciences and Veterinary Public Health, Swedish University of Agricultural Sciences, Uppsala, Sweden; 3 Department of Microbiology, National Veterinary Institute (SVA), Uppsala, Sweden; 4 Advanced Data Analysis Centre (ADAC), University of Nottingham, United Kingdom

## Abstract

Ovine footrot is a highly prevalent bacterial disease caused by *Dichelobacter nodosus* and characterised by the separation of the hoof horn from the underlying skin. The role of innate immune molecules and other bacterial communities in the development of footrot lesions remains unclear. This study shows a significant association between the high expression of IL1β and high *D. nodosus* load in footrot samples. Investigation of the microbial population identified distinct bacterial populations in the different disease stages and also depending on the level of inflammation. *Treponema* (34%), *Mycoplasma* (29%) and *Porphyromonas* (15%) were the most abundant genera associated with high levels of inflammation in footrot. In contrast, *Acinetobacter* (25%), *Corynebacteria* (17%) and *Flavobacterium* (17%) were the most abundant genera associated with high levels of inflammation in healthy feet. This demonstrates for the first time there is a distinct microbial community associated with footrot and high cytokine expression.

Footrot is an infectious disease and a major cause of lameness affecting sheep welfare worldwide^1^,^2^. The disease causes a significant financial impact, estimated to be £24 to £80 million in annual losses within the United Kingdom alone^2^. Footrot is the progression of interdigital dermatitis (ID) due to the presence and effect of *D. nodosus*. ID is characterised by inflammation of the superficial epidermal layers, which slough off irregularly. This potentially leads to underrunning footrot, characterised by the separation of the hoof horn from the sensitive underlying tissue^1^.

Virulent *D. nodosus,* a Gram negative bacterium, is essential to initiate underrunning footrot lesions^3^,^4^. *Fusobacterium necrophorum,* also a Gram negative bacterium, is attributed to play a role in the initiation of ID prior to *D. nodosus* infection^1^, and/or in the exacerbation of footrot lesions once it is established^5^. Other bacteria such as *Treponema* have also been detected in footrot cases, but its role in the disease process remains unclear^6^,^7^,^8^. A microbiota study, using a pyrosequencing approach, found 27 different bacterial genera in the ovine interdigital skin where *Peptostreptococcus* was the most abundant genus in healthy interdigital skin, *Corynebacterium* was mainly associated with ID and *Staphylococcus* was the most abundant read genus in footrot samples^6^. A similar disease in cattle, bovine digital dermatitis (BDD), also presents a complex aetiology and is considered a polymicrobial disease^9^,^10^. *Treponema* are the predominantly identified microorganism with *Fusobacterium necrophorum, Porphyromonas, Bacteroides, Campylobacter, Guggenheimella*, and *D. nodosus* also present in these lesions^9^,^10^,^11^,^12^. Research on footrot has been mainly focused on bacterial colonisation targeting *D. nodosus* and *F. necrophorum* to understand virulence mechanisms and disease progression. However, little is known about the role of other bacterial groups within skin or the ovine immune response to footrot, which may also play a fundamental role in the disease process. Advice regarding prevention of footrot in the U.K. is covered by the Agriculture and Horticulture Development Board Five point plan for tackling lameness in sheep, which includes the use of the Footvax vaccination^13^. This vaccine alone is not a standalone preventative measure and has to be used in coordination with the remaining four aspects of the suggested Five point plan^14^. Also, the efficacy of this multivalent vaccine is often questioned due to antigenic competition^14^,^15^ and limited uptake is often attributed to additional cost and time factors for farmers^2^. Current treatment of footrot is limited to parenteral antibiotics and topical bactericides^16^. Information on the immune response is an essential step for the establishment of new methods for prevention and control of footrot including the design of an efficacious and affordable vaccine. Several pro-inflammatory cytokines and chemokines are expressed in the skin including IL6, CXCL8, IL1β^17^ and IL17^18^. IL1β has a range of stimulatory effects on immune cells playing a key role in the innate immunity of the skin^19^. Similarly, IL6 is involved in key immune processes such as monocyte and T-cell recruitment, survival and differentiation^20^, and IL17 leads to release of pro-inflammatory cytokines and immune cell recruitment^18^. CXCL8 is a classical chemokine involved in the recruitment of neutrophils and proliferation of keratinocytes^21^,^22^. In the ovine interdigital space, the mRNA expression of TLR2, TLR4 and IL1β was found to be significantly elevated in severe ID and footrot^23^. Other studies so far have been focused on the investigation of the systemic ovine inflammatory response to footrot in the context of vaccination or experimental infection with *D. nodosus*^24^,^25^,^26^. Following experimental infection with *D. nodosus* and vaccination no changes in TLR4 mRNA expression in peripheral blood leukocytes was observed; in contrast, TLR2 mRNA expression levels increased in peripheral blood in leukocytes response to both, infection or vaccination^27^. In this context, we hypothesise that the pathology of footrot is a host mediated expression of local immune responses, in association with bacterial colonisation, leading to severe inflammation that can progress to underrunning footrot. This study aimed to investigate whether high virulent *D. nodosus* load and abundance of bacterial populations are associated with high levels of inflammation in the ovine interdigital skin.

## Results

### Identification of reliable reference genes for the normalization of RT-qPCR data from the ovine interdigital skin

To improve robustness of the reference gene identified in our previous study^23^, we increased sample size for each clinical condition (10 healthy, 10 mild ID, 10 moderate/severe ID and 10 footrot), the number of potential reference genes included and multiple algorithms to assess stability. In addition, we also included a co-regulation analysis. mRNA expression levels of *ACTB, GAPDH, TUBA, PPIA, 18S rRNA, TMEM79, ASCC2, C3ORF58, BHLHE40* and *DDX54* were examined in all 40 biopsies by RT-qPCR to verify their reliability as potential reference genes. The amplification efficiencies were between 90–105%, except for *TUBA* (122%) (Supplementary Table 1). The RefFinder web based tool^28^ was used to analyse gene expression stability in accordance with the MIQE guidelines^29^,^30^. Comprehensive analysis identified that *ACTB, DDX54* and *PPIA* were the most stably expressed genes and *TUBA, 18S rRNA and GAPDH* were ranked as the least stably expressed genes (Supplementary Table 2). The four algorithms incorporated by RefFinder showed slightly different gene ranking orders individually; however, *ACTB, PPIA* and *DDX54* were consistently ranked as the most stably expressed genes in all algorithms (Supplementary Table 2) with no major differences among the different clinical conditions observed. The co-regulation analysis showed that two groups of the selected reference genes had common upstream regulators: (1) *C3ORF58* and *DDX54* and (2) *ACTB, GAPDH* & *TUBA.* No direct co-regulator was known between *ACTB, PPIA* and *DDX54*, which were identified as reliably stable genes by RefFinder software.

**Figure 1:**
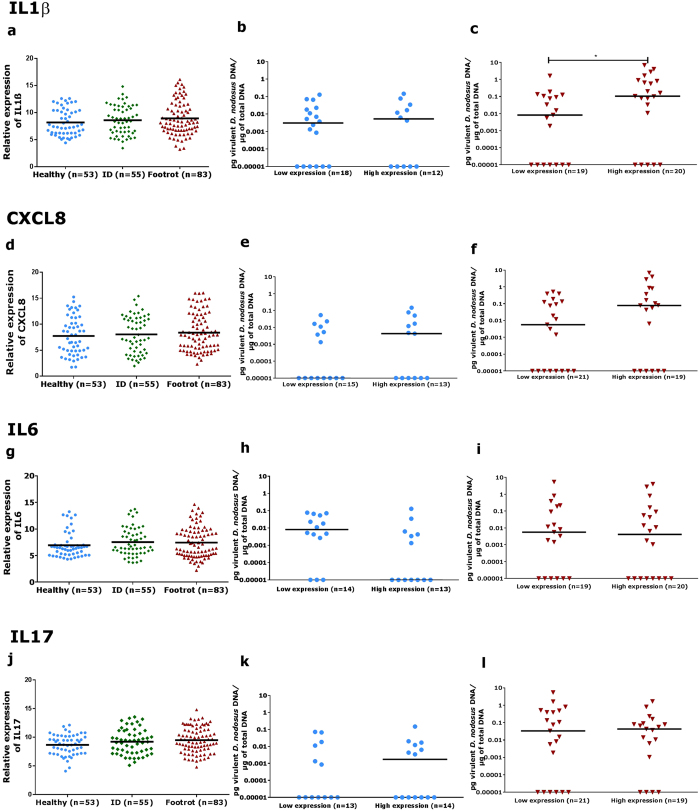
Relative mRNA expression levels of IL1β (**a**), CXCL8 (**d**), IL6 (**g**) and IL17 (**j**) in healthy, interdigital dermatitis (ID) and footrot biopsies from the ovine interdigital skin. Association between virulent *Dichelobacter nodosus* load and relative mRNA expression of IL1β (**b**), CXCL8 (**e**), IL6 (**h**) and IL17 (**k**) in healthy samples with low and high expression levels of these cytokines (blue symbols). Association between virulent *D. nodosus* load and relative mRNA expression of IL1β (**c**), CXCL8 (**f**), IL6 (**i**) and IL17 (**l**) in footrot samples with low and high expression levels of these cytokines (red symbols). Median is represented by black bars. Kruskal-Wallis test (Mann Whitney test, non-parametric) was performed to verify the association between virulent *D. nodosus* load and expression of IL1β, IL6, CXCL8 and IL17. Kruskal-Wallis test (Dunn’s multiple comparison test, non-parametric) was used to verify the association between cytokines relative mRNA expression levels with clinical conditions, *p < 0.037.

To investigate the host immune response to interdigital dermatitis and footrot, the mRNA expression levels of IL1β, IL6, CXCL8 and IL17 were analysed in 53 visually healthy, 55 ID samples and 83 footrot samples. PCR products were confirmed by sequencing and all targets showed amplification efficiencies within the ranges of an optimised RT-qPCR assay (90–105%, Supplementary Table 1). Expression of all targets was detected. Surprisingly, healthy samples showed a wide and marked inflammatory background and no differential gene expression of any inflammatory mediator in the context of clinical conditions was identified (Fig. 1a,d,g,j). This suggests that expression of the cytokines targeted in this study is not affected by ID and footrot alone, but may relate to bacterial colonisation or other non-infectious causes. In this context, we investigated whether foot conformation and integrity could affect the expression levels of cytokines in the ovine interdigital skin. There was no difference in the mRNA expression levels of IL1β, IL6 IL17 and CXCL8 in samples from feet with conformation scores 2 and 3 (Supplementary Fig. 1).

### Microbial diversity associated with disease condition

**Figure 2:**
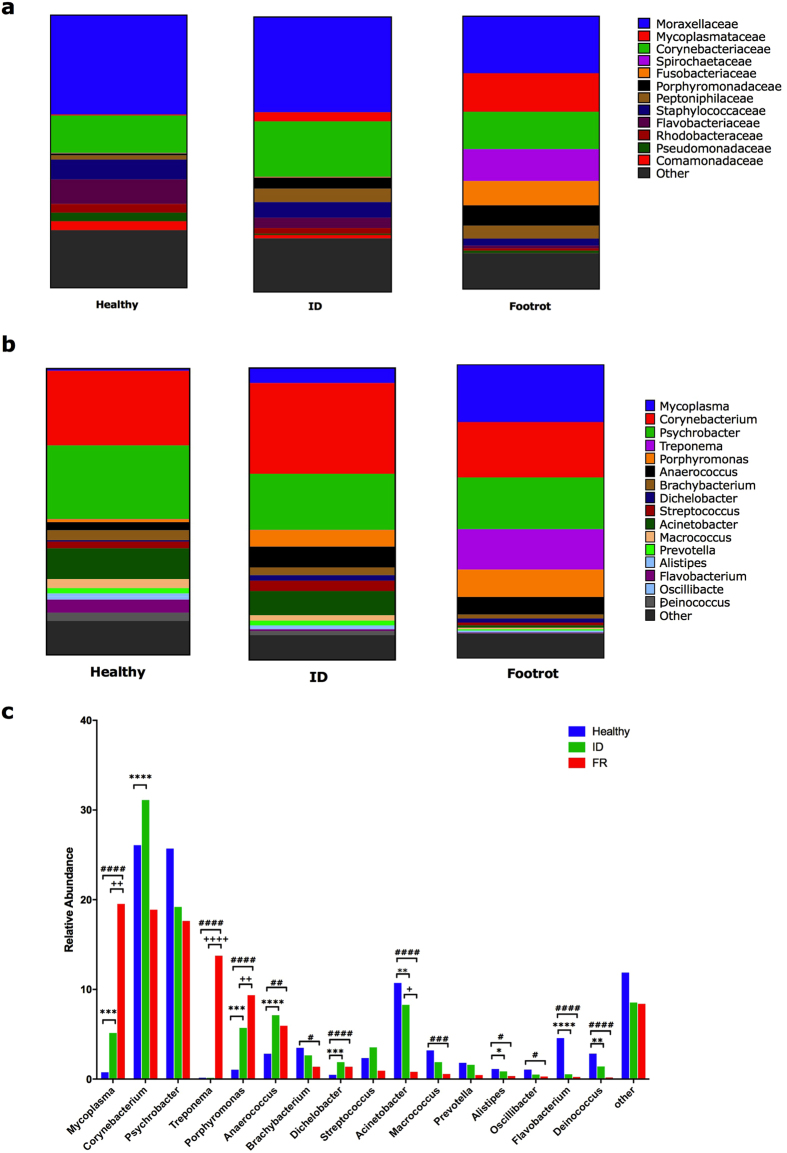
Microbial communities in ovine interdigital skin tissues from healthy, interdigital dermatitis (ID) and footrot affected feet. Relative abundance of taxonomic bacterial groups in healthy (n = 40), ID (n = 30) and footrot (n = 36) affected feet using 16 S rRNA gene based metagenomics. (**a**) at the family level (**b**) at the genus level, (**c**) at genus level with ≥1 relative abundance. ‘ID’ significantly different to ‘Healthy’ *p < 0.05, **p < 0.01, ***p < 0.001, ****P < 0.0001; ‘FR ‘ significantly different to ‘Healthy’ ^#^p < 0.05, ^##^p < 0.01, ^####^p < 0.0001; ‘FR’ significantly different to ‘ID ’ ^++^P < 0.01, ^+++^P < 0.001, ^++++^P < 0.0001.

The microbial population analysis was carried out on tissue biopsies from 40 healthy, 30 ID and 36 footrot samples. The healthy pool of samples contained 10,655,297 sequences, ID had 7,606,638 and footrot had 11,103,063. Post quality filtering the healthy pool of samples contained 8,609,842 (81%) sequences, ID had 6,625,304 (87%) and footrot had 8,999,482 (81%). After taxonomic classification using the Ribosomal database project RDPTools classifier^31^ 6,734,875 (78%) sequences were classified for healthy samples, 4,474,178 (68%) for ID and 6,030,053 (67%) for footrot (Supplementary Fig. 2). Pielou’s evenness test showed a significant difference between healthy and footrot affected samples at each taxonomic level assessed (phylum p = ≤0.0001, family p = ≤0.05 and genus p = ≤0.0001), and a significant difference between healthy and ID at phylum (p = ≤0.01) and genus (p = ≤0.01) levels. The Shannon index showed a significant difference at the genus level between healthy and footrot affected samples (p = ≤0.01) (Supplementary Fig. 3). The two main bacterial families across all three disease states were Moraxellaceae (20–36%) and Corynebacteriacae (14–20%) (Fig. 2a). The main bacterial families significantly increased in abundance in footrot compared to both, healthy and ID samples, were Mycoplasmatacae (p < 0.0001 for both), Spirochaetaceae (p < 0.0001 for both) and Fusobacteriacae (p < 0.0001 for both). Bacterial families with significantly increased abundance in healthy samples compared to footrot included Flavobacteriaceae (p < 0.0001) and Staphylococcaceae (p = 0.017) (Fig. 2a). At genus level the three most abundant bacterial genera in healthy and ID samples were *Corynebacterium* (H: 26%, ID: 31%), *Psychrobacter* (H: 26%, ID: 19%) and *Acinetobacter* (H: 11%, ID: 8%). In contrast, in footrot samples, four genera were highly represented: *Mycoplasma* (20%, p = 0.0009 vs healthy, p = 0.007 vs ID), *Corynebacterium* (19%), *Psychrobacter* (18%) and *Treponema* (14%, p < 0.0001 vs both, H and ID) (Fig. 2b,c). As expected, *D. nodosus* abundance was low (0.5–1.9%) but significantly higher in ID (p = 0.0005) and footrot samples (p < 0.0001) compared to healthy samples (Fig. 2c). Taken together the microbial community of tissue samples from the interdigital space of footrot affected feet is significantly different to that of healthy and ID affected feet.

### Association of the host immune response and virulent *D. nodosus* colonisation

Considering that clinical conditions and foot conformation were not significantly associated with higher expression of the investigated pro-inflammatory cytokines, we investigated whether the load of the causative bacterium of underrunning footrot, virulent *D. nodosus*, is associated with high expression of inflammatory markers. Low but significant correlations of virulent *D. nodosus* load and cytokine mRNA expression were identified for IL1ß (p = 0.039) and CXCL8 (p = 0.046) in samples from footrot affected feet (Table 1). To investigate this further, samples with IL1β, IL6, CXCL8 and IL17 expression values within the 25^th^ (low) and 75^th^ (high) quartiles were selected to investigate the *D. nodosus* load (Fig. 1b,c,e,f,h,i,k,l). While in healthy samples the *D. nodosus* load is similar in the low and high inflammation groups, in footrot samples with high levels of IL1β expressions also have significantly higher *D. nodosus* load (Fig. 1b,c).

### Microbial diversity associated with low and high levels of inflammation in the ovine interdigital skin

**Figure 3:**
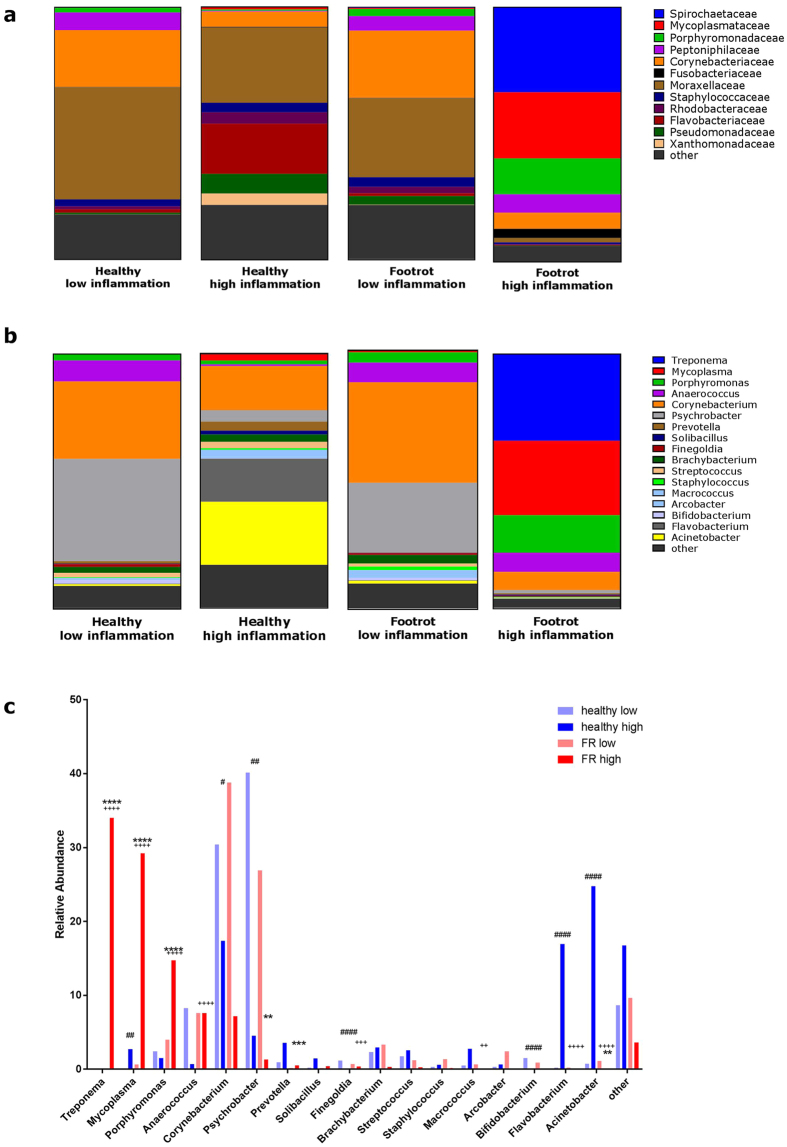
Microbial communities in ovine interdigital skin tissues with high and low levels of inflammation from healthy and footrot affected feet. Relative abundance of taxonomic bacterial groups in tissue with low and high levels of inflammation from healthy (H, n = 12 and n = 9) and footrot (FR, n = 7 and n = 8) affected feet, respectively using a 16 S rRNA amplicon survey. (**a**) at the family level (**b**) at the genus level, (**c**) at genus level with ≥1 relative abundance. ‘FR high’ significantly different to ‘FR low’ **p < 0.01, ***p < 0.001, ****P < 0.0001; ‘H high’ significantly different to ‘H low’ ^#^p < 0.05, ^##^p < 0.01, ^####^p < 0.0001; + ‘FR high’ significantly different to ‘H high’ ^++^P < 0.01, ^+++^P < 0.001, ^++++^P < 0.0001.

In the context of footrot being a polymicrobial infection and the main microorganism, *D. nodosus,* being significantly associated with high levels of IL1β in footrot samples, we further investigated whether the microbial community composition differs depending on high (75^th^ quartile) and low (25^th^ quartile) levels of inflammation using IL1β expression as a marker of inflammation. Sequence reads from the previous analysis were pooled according to IL1β expression level and corresponding disease state. A total of 21 samples were associated with healthy animals (12 low IL1β expression and 9 high IL1β expression) and 15 footrot affected samples (7 low IL1β expression and 8 high IL1β expression). The number of sequence reads were 2,273,223 and 2,699,979 for healthy low and high IL1β expression and 2,536,826 and 3,671,436 for footrot low and high respectively. After quality filtering, there were 2,032,217 (89%) and 2,182,260 (81%) for healthy low and high IL1β expression and 1,904,295 (75%) and 2,826,028 (77%) for footrot low and high, respectively. Post taxonomic classification, using the Ribosomal database project RDPTools classifier^31^, there were 1,860,595 (92%) designation for healthy low samples, 1,725,874 (79%) for healthy high, 1,783,436 (94%) for footrot low and 1,650,951 (58%) for footrot high (for individual samples see Supplementary Fig. 2). Pielou’s evenness test showed a significant difference between healthy samples with low IL1β expression and footrot affected samples with high IL1β expression (phylum p = ≤0.001 and genus p = ≤0.05) and between healthy sample with low IL1β expression and healthy samples with high IL1β expression (phylum p = ≤0.05) and footrot affected with low IL1β expression and footrot affect with high IL1β expression (phylum p = ≤0.05 and genus p = ≤0.05). Shannon index also highlighted a significant difference between healthy samples with low IL1β expression and footrot affected samples with high IL1β expression (phylum p = ≤0.01). There were no significant differences at the family taxonomic level (Supplementary Fig. 4). Surprisingly, at the taxonomic levels investigated the bacterial communities from low inflammation healthy and low inflammation footrot samples were very similar (Fig. 3a–c). On family level the main groups were Moraxellaceae (31–44% abundance) and Corynebacteriacae (23–27% abundance) (Fig. 3b); and at genus level the three most abundant bacterial genera were *Psychrobacter* (27–40%), *Corynebacterium* (30–39%) and *Anaerococcus* (8%) (Fig. 3c,d). In contrast, the microbial communities from high inflammation healthy and high inflammation footrot are very different to each other as well as to the low inflammation samples from the same disease state (Fig. 3a–c). Comparing those two microbial communities, on family level the highly abundant groups in high inflammation healthy samples are Moraxellaceae (30%) and Flavobacteriaecae (20%, p = 0.001), and in high inflammation footrot samples are Spirochaetaceae (33%, p < 0.0001), Mycoplasmataecae (26%, p = 0.0002) and Porphyromonadaecae (14%, p < 0.0001) (Fig. 3b). At genus level the most abundant genera in high inflammation healthy samples were *Acinetobacter* (25%), *Corynebacteria* (17%) and *Flavobacterium* (17%), which are all significantly less abundant in low inflammation healthy samples (p < 0.0001, p = 0.035 and p < 0.0001, respectively) (Fig. 3c,d). In high inflammation footrot samples, *Acinetobacter* (0.05%) and *Flavobacterium* (0.05%) are significantly less abundant (p < 0.0001 for both) (Fig. 3c,d). In contrast, the most abundant genera in those high inflammation footrot samples are *Treponema* (34%), *Mycoplasma* (29%) and *Porphyromonas* (15%), which are significantly less abundant in both, low inflammation footrot samples (p < 0.0001, p < 0.0001, p = 0.0006, p = 0.039, respectively) and high inflammation healthy samples (p < 0.0001 for all 4 genera) (Fig. 3c,d). Taken together this demonstrates while the bacteria communities are very similar in low inflammation healthy and footrot samples, high inflammation samples from healthy as well as from footrot samples have distinct and very different bacterial communities.

## Discussion

This study investigated the microbial community in the context of inflammation in healthy and footrot affected ovine feet and showed for the first time that inflammation, as marked by high mRNA expression levels of IL1β, a central mediator of immune response in the skin, is associated with a distinct microbial community in the ovine interdigital skin. Analysis of the microbial community showed distinct bacterial populations in tissues from healthy, ID and footrot affected feet. The four most abundant phyla in all skin samples, irrespective of clinical conditions, were Actinobaceria, Firmicutes, Proteobacteria and Bacteriodetes, which were also found to be the most abundant phyla in the interdigital space of healthy dogs^32^ and in healthy bovine skin of the claw area^10^,^33^. The same phyla were found in bovine DD lesions^33^ at differing proportions, whilst Tenericutes, Spirochaetes and Fusobacteria are enriched in footrot samples. In healthy and ID feet the most abundant genera were *Corynebacteria, Psychrobacter* and *Acinetobacter*, while in footrot the most abundant genera were *Mycoplasma, Corynebacteria, Psychrobacter* and *Treponema*. However, these populations differ from a small study that was the first publication of the 16S based ovine foot microbiome, where *Macrococcus, Peptostreptococcus* and *Corynebacteria* were most abundant in healthy, *Macrococcus* and *Corynebacteria* were most abundant in ID and *Macrococcus, Staphylococcus* and *Corynebacteria* most abundant in footrot affected feet^6^. There are several factors that explain those differences, in our study the sample number was higher (40 healthy, 30 ID and 36 footrot versus 3 healthy, 2 ID, 1 footrot), each of our samples was from an individual foot and not pooled from all feet per sheep. This could affect the footrot specific community identified if the individual animal only had one foot affected. In addition, the sequencing technology and data processing pipelines have significantly changed in the last few years. As expected, *D. nodosus* had greater abundance in footrot samples as was also shown by qPCR to have a higher load. Surprisingly, the microbial community composition appears to show a stronger correlation with inflammation level rather than disease state since there is very little difference between the microbiome of healthy and footrot low inflammation biopsies while the microbial community and the dominant genera differed greatly between healthy high inflammation (*Acinetobacter, Corynebacterium, Flavobacterium*) and footrot high inflammation tissues (*Treponema, Mycoplasma, Porphyromonas*). Increased proportions of *Corynebacteria* were also observed in canine atopic dermatitis^34^. Of the bacterial genera identified, *Dichelobacter, Fusobacteria* and *Treponema* all linked to ovine or bovine foot disease^4^,^6^,^7^,^8^,^10^. *Prevotella* and *Porphyromonas* were isolated from footrot cases in goats^35^ and were recently reclassified from *Bacteriodes melaninogenicus*^36^, a species regularly isolated from bovine interdigital necrobacillosis (bovine footrot)^37^. *Corynebacterium*, a non-motile and facultative anaerobic bacterium, has been reported to be abundant in tissue biopsies from ID affected feet^6^, but also near the surface of the interdigital skin of sheep and in footrot lesions^38^. In addition, *Corynebacterium* has been found in ovine heel abscesses^39^. *Psychrobacter* species are rarely associated with disease in animals. Two subspecies have been isolated from congested lungs from two different lambs (ages 20 days & 1 year) after sudden death^40^. *Mycoplasma* species are linked to lameness, generally associated with polyarthritis in a wide range of animals, mainly pigs and poultry^41^,^42^, but also cattle^43^,^44^ and occasionally in sheep^45^.

The methodology we used, a 16S rRNA amplicon survey, has some inherent limitations associated with the data generated and the way in which they are analysed and how the results are interpreted. However, through experimental design and robust data analysis tools we have reduced those limitations where possible. While metagenomic whole genome sequencing is often the favoured methodology, with in-tissue samples like our interdigital post slaughter biopsies, microbial DNA typically accounts for less than 1% of the total DNA yield and methods of enrichment are required as host DNA would greatly overwhelm the microbial DNA present^46^. This is normally achieved by either host DNA depletion, increased sequencing depth or microbial enrichment like 16S rRNA amplification. In this situation, an amplicon approach is preferred because the required increase in sequencing depth would be cost prohibitive and current methods of host DNA depletion are more suited to biological fluid samples, where the host to microbe DNA ratio is more favourable. Short read sequencing requires targeting of a variable region. This may introduce bias due to poor resolution between bacteria^47^, under representation of some genera^48^ and positive selection for others^49^,^50^. Here the V3/V4 region was used as they are incorporated into the Illumina library prep workflow and are suggested to be a good primer pair for 16S rRNA discrimination^51^. However, this paring may not be the most optimal, and V4/V6 has now been shown to provide superior resolution^50^. Recently, contamination issues have been identified relating to the laboratory environment, reagents and preparation kit^52^. It is therefore important to be aware that some contaminants may be present in the data set, however most are common water or soil bacteria, which in context of this study it seems drastic to discount their presence completely as damaged interdigital skin is in close contact with soil and water. A pragmatic approach must be used to determine if their presence could be of any importance and abundance calculations used with some scepticism. Sequencing read number and quantity of input DNA need to be taken into account as low numbers of reads and low quality DNA can artificially exaggerate abundance within a sample due to poor amplification^53^. All DNA extractions used in this study were compared for uniformity and have average quantity of bacterial DNA; healthy samples 0.12 ng/μl (SD 0.7), ID 0.08 ng/μl (SD 0.5) and footrot 0.17 ng/μl (SD 3.6).

The host immune response was investigated through the mRNA expression of a panel of six inflammatory genes likely to have a role in skin immune responses in sheep and cattle^23^,^54^,^55^. Surprisingly, in the context of foot disease, no differential expression of IL1β, IL6, CXCL8 and IL17 was identified in ID and footrot compared to healthy samples. Using a smaller sample set (n = 28), our previous work has shown IL1β to be elevated in severe ID and footrot in relation to healthy samples^23^. These divergent results can be associated with a higher expression of IL1β in healthy samples found in this study than was observed by Davenport *et al*.^23^. Samples for the present study were collected between autumn and winter and it was noted that the weather had been significantly wetter than when samples were collected for the previous study. Wetter environmental conditions have been shown to increase the prevalence of interdigital dermatitis and footrot^15^,^38^,^39^,^56^,^57^, therefore this might also impact on the basal levels of inflammation and may favour inflammation in the interdigital skin of apparently healthy samples. The wide inflammatory background seen in healthy samples might also be associated with the presence of subclinical disease that may have developed into ID and footrot in the next few days. However, using abattoir samples makes it impossible to investigate the progression of the disease and thus verify whether healthy feet would develop footrot lesions. These sheep might also have had footrot previously and the wide inflammatory response could be related to a healing process.

Infectious as well as non-infectious factors might be associated with inflammatory responses in the skin. Particularly in the context of footrot, poor foot conformation could be a non-infectious factor leading to a higher expression of inflammatory genes indicating underlying damage and disease. Sheep with damaged sole and heel area have an increased susceptibility to become lame and consequently develop footrot^16^. In the present study, we showed that the effects of poor foot conformation were not directly associated with a higher inflammatory response, since slightly higher but not significant mRNA expression levels of cytokines was present in feet with poor conformation.

A significant association between samples with high expression of IL1β and high *D. nodosus* load in footrot samples as well as significant correlations between CXCL8 and IL1β with *D. nodosus* in footrot samples, but not in healthy samples, further confirmed that *D. nodosus* infection could play a key role in the development of an inflammatory microenvironment characterised by those pro-inflammatory cytokines. It is important to highlight that based on these experiments, it is not possible to conclude whether *D. nodosus* triggers the expression of CXCL8 and IL1β or the inflammation in the skin may facilitate the invasion of this bacterium. In our previous study, IL1β levels were elevated in ovine dermal fibroblasts stimulated with LPS or heat-killed extracts of *D. nodosus*, suggesting that both IL1β and fibroblasts play a key role in the host response to footrot^23^. CXCL8 production is part of the initial immune response and plays a role in the recruitment of neutrophils, as well as having an effect on the proliferation of keratinocytes^22^. Keratinocytes are key cells synthesising the potent pro-inflammatory cytokine IL1β^58^. This may explain the thickened epidermal layer of ovine interdigital skin observed in histological studies of footrot^1^. Both cytokines, CXCL8 and IL1β, were highly expressed in sheep with skin pyogranulomas^59^, in response to inflamed foetal ovine skin^60^ and also in bovine digital dermatitis lesions in cattle feet^54^.

In summary, this study has shown distinct dysbiosis evident across disease states and the association with inflammation, highlighting the importance of the microbial population in relation to the local inflammatory response within the context of the healthy or footrot affected ovine foot.

## Material and Methods

### Study design and sample collection

Ovine interdigital post-slaughter biopsies were collected at an abattoir (Leicestershire, U.K.) at various time points (Oct 2013-Jan 2015) using a convenience sampling approach due to variable availability of the clinical conditions at slaughter. Firstly, ovine feet were scored for conformation/integrity of the sole and heel/wall of each digit: 0 = undamaged sole and heel area with a perfect shape. 1 = mildly damaged/misshapen sole and/or heel area of the digit (<25%). 2 = moderately damaged/misshapen sole and/or heel area of the digit (>25% and <75%). 3 = severely damaged/misshapen sole and/or heel area of the digit (>75%). Then, ovine feet disease status was scored as described previously^16^, allowing classification into healthy, ID or footrot feet according to established scoring criteria: absence of interdigital skin lesion = healthy; slight interdigital skin lesion (≤5% affected) = mild ID; moderate to severe ID lesion (>5% affected); presence of underrunning lesion = footrot^8^. The sample set (n = 191) included 53 healthy, 55 interdigital dermatitis (ID) and 83 footrot biopsy samples. Biopsies were collected using disposable 6mm biopsy punches (National Veterinary Services) and then stored in 2 ml of RNALater^®^ (Sigma-Aldrich, St. Luis, USA) according to manufacturer’s instructions. Each biopsy was cut in half; one piece was used for DNA extraction and the other one for RNA extraction.

### Quantification of virulent *D. nodosus*

Tissue homogenisation and DNA extractions were performed as described previously using QIAamp cador^®^ kit (QIAGEN, Hilden, Germany)^8^. Virulent *D. nodosus* was quantified based on the presence of *aprV2* gene by quantitative PCR in an Applied Biosystems^®^ 7500 Fast Real-Time PCR System (Thermo Fisher Scientific Inc., Waltham, USA) as previously described^7^. The data were normalised as pg of virulent *D. nodosus* DNA present in the total μg of DNA extracted from each tissue biopsy.

### Microbial Diversity

Extracted DNA was subjected to a 16S rRNA V3/V4 variable region amplification by standard PCR using the Illumina metagenomic library preparation kit and primers 341F and 534R (Supplementary Table 3). The libraries were sequenced using an Illumina MiSeq at the National Veterinary Institute (SVA), Sweden. Sequencing consisted of four 2 × 300 bp runs, resulting in approximately 33 Gb of data (All raw sequence reads are available in the NCBI SRA Project number PRJNA369597). The generated reads where parsed through Trimmomatic (version 0.36)^61^ to remove Illumina sequencing adaptors and 16S rRNA primer contamination based on Klindworth *et al*.^62^. Remaining sequences were also assessed and filtered using a 15 bp sliding window approach for quality scores (phred >30) and were required to be of a minimum length of 50 bp. The trimmed high quality reads where analysed using a local installation of the RDPTools 16S supervised taxonomic classification workflow^31^ (https://github.com/rdpstaff/classifier). Briefly, UCHIME^63^ was used to identify putative chimeric reads to be removed from the quality trimmed sequence file. Taxonomy was then assigned using the naïve Bayesian RDP classifier^31^, with the default assignment cutoff of 0.8 and with the 16S SSU rRNA gene copy number adjustment enabled. Based on the qPCR data, the assigned samples were concatenated in 25^th^ and 75^th^ quartiles of IL1β expression for healthy and footrot affected samples allowing for population comparison. The counts (normalised for 16S rRNA copy number) per taxonomic level were imported into R for analysis using Vegan and Fossil packages^64^,^65^. Microbial diversity at phylum, family and genus taxonomic levels were assessed by calculating the Shannon index and Pielou’s Evenness test.

### RNA extraction & cDNA synthesis

Half biopsies were homogenised as described previously^23^. RNA was isolated using NucleoSpin RNA isolation kit (Machery-Nagel, Germany) following the manufacturer’s recommendations. RNA concentration and quality were analysed using RNA 6000 Nano Kit Bioanalyser (Agilent technologies, Germany). RNA was diluted in water and cDNA was synthesised using M-MLV Reverse Transcriptase (Promega, Madison, USA) according to manufacturer’s instructions. The final volume of each reaction was diluted in RNAse/DNAse free water (Fisher Scientific, UK).

### Quantification of ovine gene expression

Ovine mRNA expression levels of β-Actin (*ACTB*), Glyceraldehyde-3-Phosphate Dehydrogenase (*GAPDH*), α-Tubulin (*TUBA*), Cyclophilin (*PPIA*) and Eukaryotic 18S ribosomal RNA (18S rRNA), Activating signal cointegrator 1 complex subunit 2 (*ASCC2*), basic helix-loop-helix family, member e40 (*BBHEL*), Transmembrane protein 79 (*TMEM79*), DEAD (Asp-Glu-Ala-Asp) box polypeptide 54 (*DDX54*) and chromosome 3 open reading frame 58 (*C3ORF58*), Interleukins IL1β, IL6, CXCL8 and IL17 *w*ere investigated by RT-qPCR using a LightCycler^®^ 480 system (Roche Applied Science, UK). Primers for *ASCC2, BBHEL, TMEM79, DDX54* and *C3ORF58* genes were designed using Primer3Plus (version 0.4.0). For each gene, a minimum of three forward and three reverse primers were designed and assessed by RT-qPCR (Supplementary Table 2). All assays were performed with manual reaction setup using the LightCycler 480 system (Roche Applied Science, UK). Reactions contained 5 μl of cDNA (1/100 dilutions) in 1 × SYBR green qPCR master mix (Sigma-Aldrich, UK) with 1 μM of forward and reverse primers (Sigma Aldrich, UK). All standard dilutions, samples and no template controls (NTC) were performed in triplicates. For *ASCC2, BBHEL, TMEM79, DDX54* and *C3ORF58*, samples were subjected to initial denaturation at 95 °C for 20 minutes, followed by 45 cycles at 95 °C for 5 seconds, 60 °C for 20 seconds and 72 °C for 30 seconds and a continuous dissociation step at 97 °C to obtain a melt curve. For *ACTB, GAPDH, TUBA, PPIA*, 18S rRNA, IL1β, IL6, CXCL8 and IL17 the *s*amples were subjected to initial denaturation at 95 °C for 10 minutes, followed by 45 cycles at 95 °C for 10 seconds, 60 °C for 50 seconds and 72 °C for 1 minute and a final dissociation step at 97 °C.

### Analysis of reference genes stability and normalisation analysis

Analysis of reference genes stability was performed using RefFinder^28^, integrating geNorm^66^, Normfinder^67^, BestKeeper^68^, and a comparative ΔCq method, to compare and rank the tested candidate reference genes^69^. Upstream regulators were identified using the Ingenuity Pathway Analysis knowledgebase (IPA^®^, QIAGEN Redwood City). Normalised expression of RT-qPCR data of cytokines was calculated using the following formula: CT + (NT-CT′) * S/S′ with S = target gene slope; S′ = reference gene slope; CT = Cq value of targeted gene for each sample; CT′ = Cq value of reference gene for each sample; NT = mean of Cq values of reference gene for all samples^70^.

### Richness and diversity analysis

The concatenated taxonomic assignment data was imported into R^71^ to calculate diversity and richness statistics. Relative abundance was used at each taxonomic level to calculate the Shannon index and Pielous evenness using the vegan package^64^ Kruskal-Wallis test (Dunn’s multiple comparison test, non-parametric) was used to assess statistical differences in the diversity statistics. All analyses were performed using GraphPad Prism^®^ (Version 6.0, La Jolla, USA) and a P-value ≤ 0.05 was considered significant. Determination of statistically significant differences in populations was carried out using R^71^ with the edgeR^72^ wrapper as part of phyloseq 1.6.1 package^73^.

## Additional Information

**How to cite this article:** Maboni, G. *et al*. A distinct bacterial dysbiosis associated skin inflammation in ovine footrot. *Sci. Rep.* **7**, 45220; doi: 10.1038/srep45220 (2017).

**Publisher's note:** Springer Nature remains neutral with regard to jurisdictional claims in published maps and institutional affiliations.

## Supplementary Material

Supplementary Figure and Tables

## Figures and Tables

**Table 1:** Correlation analysis between pro-inflammatory cytokines relative mRNA expression and virulent *Dichelobacter nodosus* load.

	IL1β vs. D. nodosus		CXCL8 vs. D. nodosus		IL6 vs. D. nodosus		IL17 vs. D. nodosus	
	r	P value	r	P value	r	P value	r	P value
Healthy	0.029	0.839	0.096	0.492	−0.042	0.764	0.202	0.152
Footrot	0.229	**0.040**	0.221	**0.046**	−0.082	0.558	0.025	0.858

Bold values: statistically significant P value (<0.05). r: Pearson’s correlation coefficient.
