# Supplementary material for: A distinct bacterial dysbiosis associated skin inflammation in ovine footrot
Source: Sci Rep. 2017 Mar 24;7:45220. doi: 10.1038/srep45220 (PMC5364556; doi:10.1038/srep45220)
Supplement: Supplementary Figure and Tables [file srep45220-s1.pdf]

A distinct bacterial dysbiosis associated skin inflammation in ovine footrot

Grazieli Maboni[a*], Adam Blanchard[a*], Sara Frosth[b,c], Ceri Stewart[a], Richard Emes[a,d] and Sabine Tötemeyer[a,e]

[a] University of Nottingham, School of Veterinary Medicine and Science, Sutton Bonington, United Kingdom

[b] Department of Biomedical Sciences and Veterinary Public Health, Swedish University of Agricultural Sciences, Uppsala, Sweden

[c] Department of Microbiology, National Veterinary Institute (SVA), Uppsala, Sweden

[d] Advanced Data Analysis Centre (ADAC), University of Nottingham, United Kingdom

[e] Email for correspondence: sabine.totemeyer@nottingham.ac.uk

* Both authors contributed equally

Supplementary Table 1. Standard curve data for RT-qPCR assays

Gene	Cq values range	Slope	R^2	Efficiency	Melt curve peaks
IL1β	23-37	-3.482	0.963	93.7%	Single
IL6	26-38	-3.321	0.709	100%	Single
CXCL8	26-32	-3.489	0.998	93.5%	Single
IL17	25-34	-3.460	0.905	94.5%	Single
18S rRNA	11 - 13	-3.423	0.998	96%	Single
ACTB	22- 29	-3.563	0.997	91%	Single
TUBA	31 - 37	-4.45	0.865	122%	Single
PPIA	23 - 49	-3.586	0.961	90%	Single
GAPDH	20 - 34	-3.464	0.870	94%	Single
TMEM79	27 - 30	-3.413	0.963	96%	Single
ASCC2	30 - 35	-3.466	0.985	94%	Single
C3ORF58	30 - 32	-3.250	0.953	103%	Single
BHLHE40	27 - 30	-3.559	0.998	91%	Single
DDX54	29 - 32	-3.410	0.997	96%	Single

ACTB: β-Actin; *PPIA*: cyclophilin; *18S rRNA*: eucariotic 18S ribossomal RNA; *TUBA*: α-tubulin; *GAPDH*: Glyceraldehyde-3-Phosphate Dehydrogenase; *TMEM79:* Transmembrane protein 79; *ASCC2:* Activating signal cointegrator 1 complex subunit 2; *C3ORF58*: chromosome 3 open reading frame 58; *BHLHE40:* basic helix-loop-helix family, member e40; *DDX54:* DEAD (Asp-Glu-Ala-Asp) box polypeptide 54. Cq= quantification cycles.

Supplementary Table 2. Gene expression stability analysis using RefFinder software. Samples from all clinical conditions were included in this analysis (n=40). Low stability values= high gene expression stability.

**Genorm**		**NormFinder**		**Bestkeeper**		**Delta CT**		**Comprehensive analysis**	
Gene	Stability value	Gene	Stability value	Gene	Stability value	Gene	Stability value	Gene	Stability value
ACTB	0.348	*ACTB*	1.414	*ACTB*	0.514	*DDX54*	1.374	*DDX54*	0.323
PPIA	0.348	*DDX54*	1.732	*PPIA*	0.534	*ACTB*	1.396	*ACTB*	0.365
DDX54	0.582	*PPIA*	2.06	*DDX54*	0.565	*PPIA*	1.413	*PPIA*	0.428
C3ORF58	0.717	*C3ORF58*	4.427	*TMEM79*	0.706	*C3ORF58*	1.488	*C3ORF58*	0.56
ASCC2	0.848	*ASCC2*	5.623	*18S rRNA*	0.723	*ASCC2*	1.627	*ASCC2*	0.857
BHLHLE40	0.995	*BHLHLE40*	6.236	*C3ORF58*	0.727	*BHLHLE40*	1.813	*BHLHLE40*	1.278
TUBA	1.16	*TMEM79*	7.348	*BHLHLE40*	0.76	*TUBA*	1.993	*TUBA*	1.499
GAPDH	1.301	*TUBA*	7.454	*ASCC2*	0.91	*GAPDH*	2.022	*GAPDH*	1.518
TMEM79	1.629	*18S rRNA*	8.409	*TUBA*	0.967	*TMEM79*	2.884	*TMEM79*	2.621
18S rRNA	1.899	*GAPDH*	8.459	*GAPDH*	1.584	*18S rRNA*	2.978	*18S rRNA*	2.712

ACTB: β-Actin; *PPIA*: cyclophilin; *18S rRNA*: eucariotic 18S ribossomal RNA; *TUBA*: α-tubulin; *GAPDH*: Glyceraldehyde-3-Phosphate Dehydrogenase; *TMEM79:* Transmembrane protein 79; *ASCC2:* Activating signal cointegrator 1 complex subunit 2; *C3ORF58*: chromosome 3 open reading frame 58; *BHLHE40:* basic helix-loop-helix family, member e40; *DDX54:* DEAD (Asp-Glu-Ala-Asp) box polypeptide 54.

Supplementary Table 3. RT-qPCR and PCR Primers

Gene	Strand	Sequence 5`-3`	Accession	Reference
*GAPDH**	Forward	CCACCAACTGCTTGGCCCCC	AF022183.1	(Davenport et al., 2014)
	Reverse	GGACACGTTGGGGGTGGGGA		
B-Actin	Forward	TGTGCGTGACATCAAGGAGAA	NM_001009784	(Hughes et al., 2011)
	Reverse	CGCAGTGGCCATCTCCTG		
18S rRNA	Forward	GCAATTATTCCCCATGAACG	DQ013885	(Taylor et al., 2008)
	Reverse	CAAAGGGCAGGGACTTAATC		
16S rRNA*	Forward 341	TCGTCGGCAGCGTCAGATGTGTATAAGA GACAGCCTACGGGNGGCWGCAG		(Klindworth et al 2013)
	Reverse 534	GTCTCGTGGGCTCGGAGATGTGTATAAG AGACAGGACTACHVGGGTATCTAATCC		
α-Tubulin	Forward	CCAGATGGTGAAATGTGACC	AF251146	(Taylor et al., 2008)
	Reverse	GCATTGACATCTTTGGGAAC		
PPIA	Forward	CATACAGGTCCTGGCATCTTGTC	AY251270	(Lloyd et al., 2012)
	Reverse	TGCCATCCAACCACTCAGTCT		
GAPDH	Forward	TCCGTTGTGGATCTGACCTG	AF030943	(Hughes et al., 2011)
	Reverse	TGCTTCACCACCTTCTTGATCTC		
ASCC2	Forward	AGGAGGCTTGAAGACAGCAA	XM_04017467	This study
	Reverse	GTTGTCACAGCTGCTTTCCA		
BHLHLE40	Forward	CTACCAGGGATGGATTTTGC	NM_001129741	This study
	Reverse	TCCGGTCACGTCTCTTTTTC		
C3ORF58	Forward	TGGTGCTACAGAGTTTTCCAT	XM_012130530	This study
	Reverse	GCGAGGTCAACTCGTTTCTC		
DDX54	Forward	GAAGCTGGGACCTGGTAGAC	XM_012098176	This study
	Reverse	GTTCTGAGCTCGCACCATCT		
TMEM79	Forward	CATTGTACTGGGGATCCTGGT	XM_004002610	This study
	Reverse	ATGAAGAGCTGGACCGACTG		
IL1β	Forward	TTCTGCATGAGCTTCGTACAA	X56972.1	(Darlay et al., 2011)
	Reverse	GGGTCGGTGTATCACCTTTTT		
IL17	Forward	GAGTCTGGTGGCTCTTGTGA	XM_004018887	This study
	Reverse	TGCTGTGGGAAGTTCTTGTC		
CXCL8	Forward	GAGAAGTCCTCTGGGACAGC	NM_001009401	This study
	Reverse	CAGCCAGCTTGGAAGTCATA		
IL6	Forward	AATTTCCTGCAGTTCAGCCT	NM_001009392	This study
	Reverse	GTTTCTGACCAGAGGAGGGA		
AprV2	*aprV2/B2F*	GAAGGCGACTGGTTTGATAACTG	M35016	(Frosth et al., 2015)
	aprV2/B2R	GAGCTGTCGCTTCTTTCTTTGC		
	aprV2probe FAM-MGBNFQ	ATGCGGTGGTTATCCT		

All primers were used for qPCR except *Primers, which were used in the conventional PCR reaction for quality control of cDNA synthesis.

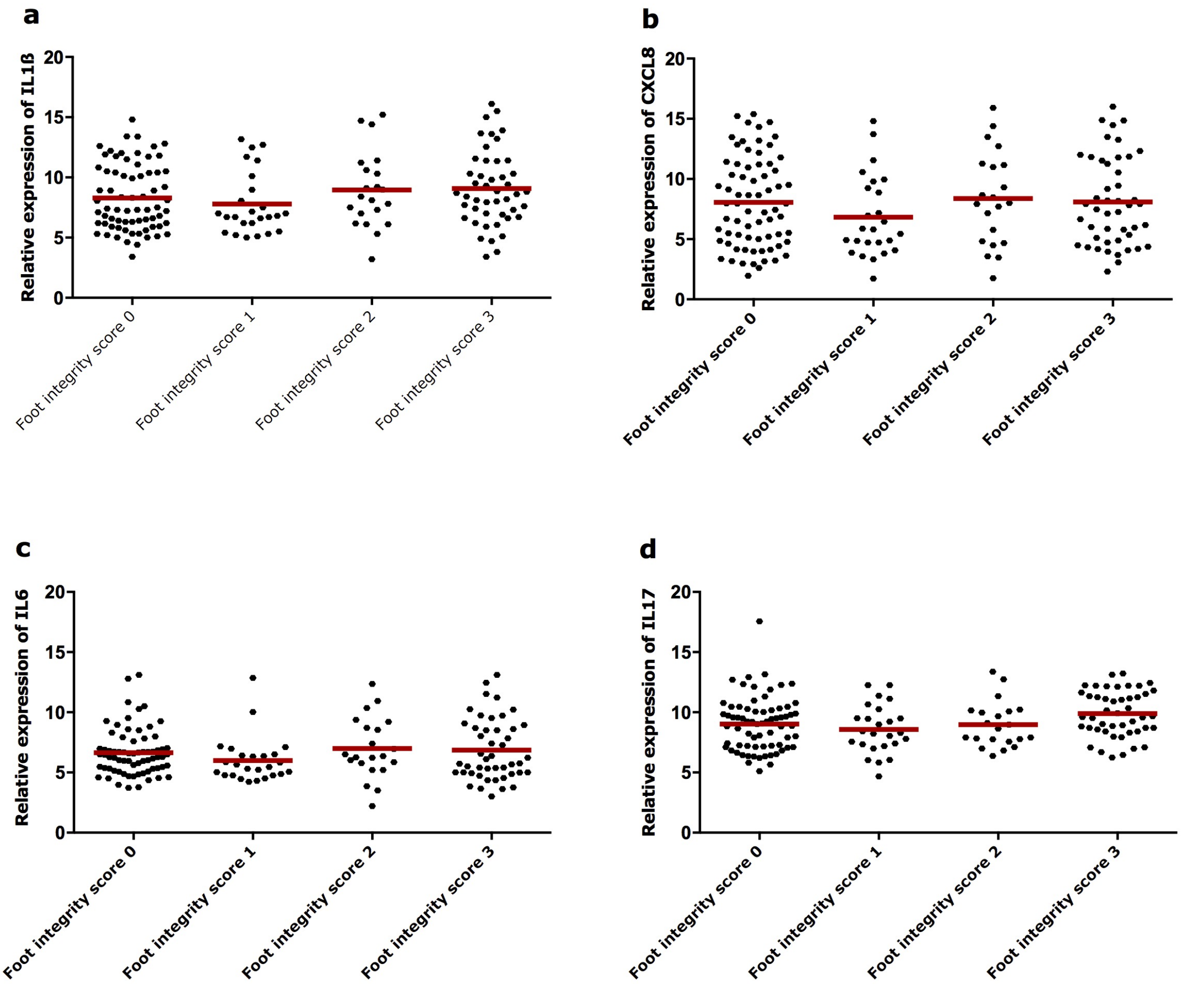

Supplementary Figure 1. **mRNA expression levels of proinflammatory cytokines across the different ovine foot conformation/integrity scores.** (a) IL1β, (b) CXCL8, (c) IL6 and (d) IL17. Ovine foot scoring system conformation of the sole and heel/wall of each digit: **0**= undamaged sole and heel area with a perfect shape. **1**= mildly damaged/misshapen sole and/or heel area of the digit (<25%). **2**= moderately damaged/misshapen sole and/or heel area of the digit (>25% and <75%). **3**= severely damaged/misshapen sole and/or heel area of the digit (>75%). Mean is represented by red bars. Data were analysed by Dunn's multiple comparisons test.

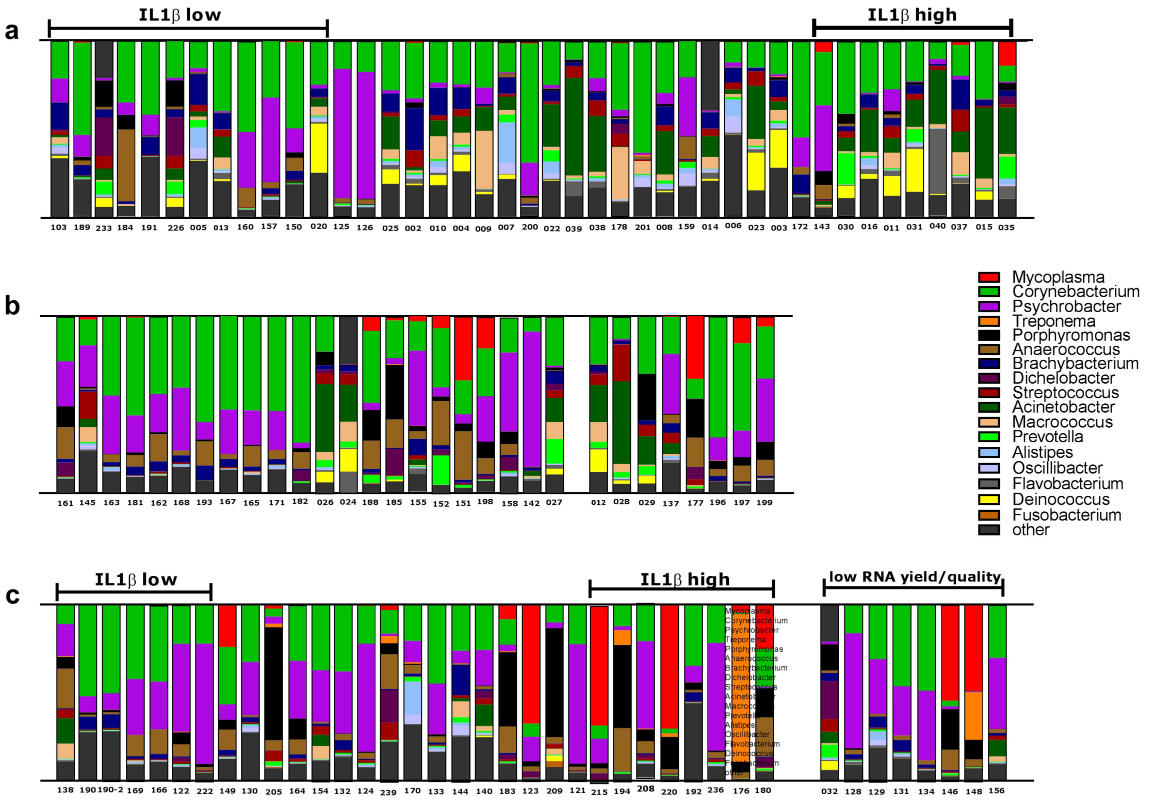

Supplementary Figure 2. Composition of individual bacterial communities. (a) Visibly healthy feet; (b) feet with interdigital dermatitis; (c) feet with footrot. Associated qPCR quantified levels of IL1β and samples with poor RNA yield are highlighted to show samples reused in further analysis and omitted due to lack of sample for qPCR analysis.

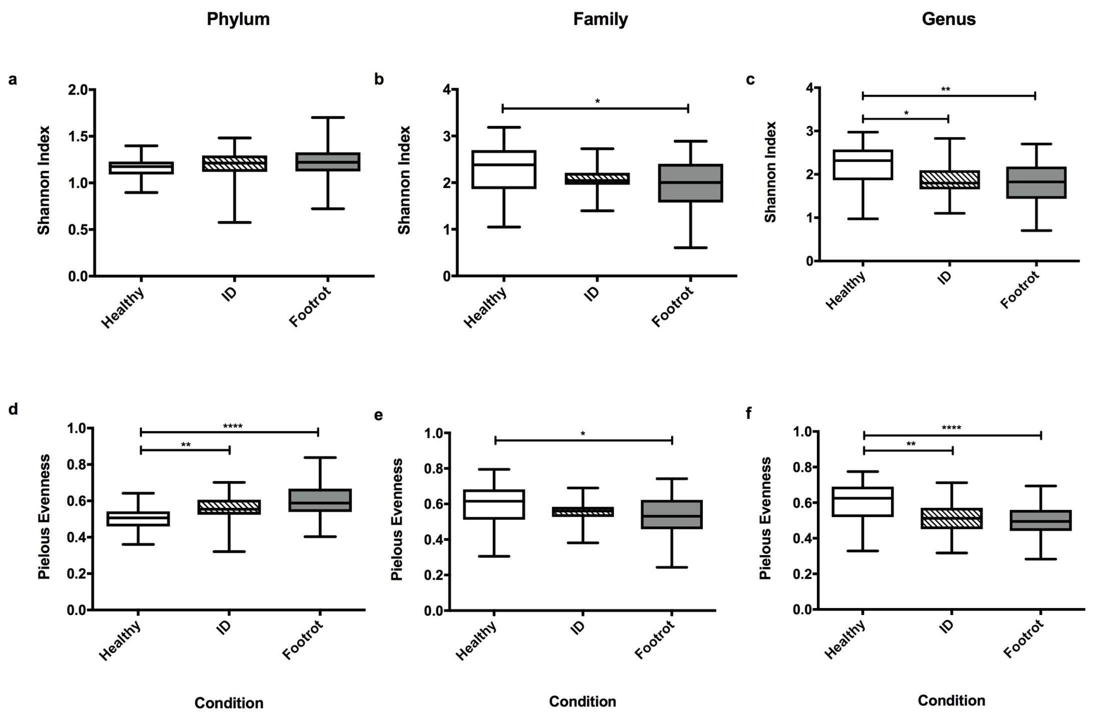

Supplementary Figure 3. Differences between diversity indices associated with disease state. Indices were calculated for each sample according to disease state and analysed for significant differences using Kruskal-Wallis test (Dunn`s multiple comparison test, non-parametric). **a,b,c** show Shannon diversity indices, **d,e,f** show Pielous evenness indices for each taxonomic level. Sample numbers are Healthy n=40, ID n=30 and Footrot n=36. P-values are displayed as * p= ≤ 0.05, ** p= ≤ 0.01, ***p= ≤ 0.001, ****p= ≤ 0.0001.

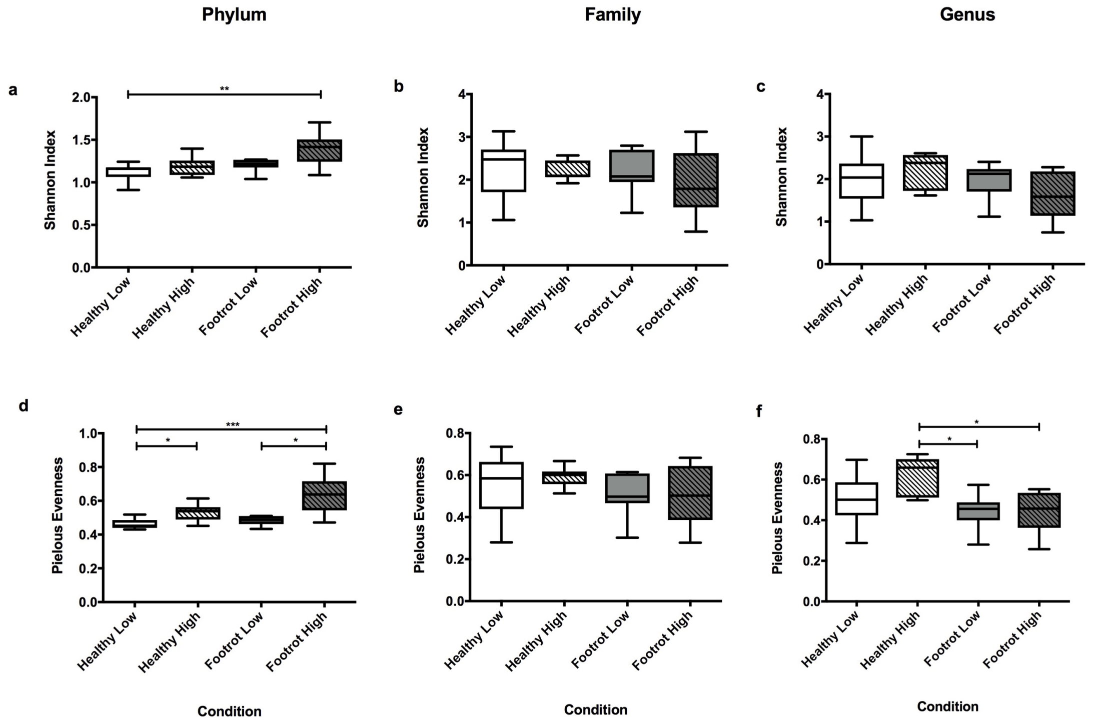

Supplementary Figure 4. Differences in diversity indices associated with inflammation and disease state. Indices were calculated for each sample according to disease state and analysed for significant differences using Kruskal-Wallis test (Dunn`s multiple comparison test, non-parametric). **a,b,c** show Shannon diversity indices, **d,e,f** show Pielous evenness indices for each taxonomic level. Sample numbers are healthy low n=12, healthy high n=9, footrot low n=7 and footrot high n=8. P-values are displayed as * p= ≤ 0.05, ** p= ≤ 0.01, ***p= ≤ 0.001, ****p= ≤ 0.0001.
